# Predictive ability of microvascular resistance reserve for periprocedural myocardial injury in patients with stable coronary artery disease undergoing percutaneous coronary intervention

**DOI:** 10.1007/s00380-025-02646-z

**Published:** 2025-12-29

**Authors:** Mikio Shigehara, Hiroki Ikenaga, Yuki Yoshitomi, Hiroshi Kobatake, Atsushi Kuraishi, Ayano Osawa, Takayuki Nakano, Yuichi Morita, Tasuku Higashihara, Noriaki Watanabe, Yoshiharu Sada, Yukiko Nakano

**Affiliations:** Department of Cardiovascular Medicine, Hiroshima University Graduate School of Biomedical and Health Sciences, 1-2-3 Kasumi, Minami-ku, Hiroshima, 734-8551 Japan

**Keywords:** Microvascular resistance reserve, Index of microcirculatory resistance, Periprocedural myocardial injury, Coronary microvascular dysfunction, Percutaneous coronary intervention

## Abstract

Periprocedural myocardial injury (PMI) has shown an association with worse prognosis after percutaneous coronary intervention (PCI). The microvascular resistance reserve (MRR) has been introduced as an index to evaluate microvascular vasodilatory capacity, independent of epicardial disease. Given the unclear association between MRR and PMI, this study aimed to determine the relationship between PMI and coronary microvascular dysfunction, including index of microcirculatory resistance (IMR) and MRR. This study identified 68 patients with stable angina pectoris who underwent PCI after measurements of coronary circulatory physiological indices at Hiroshima University Hospital between October 2017 and October 2024. PMI was assessed by a post-PCI high-sensitivity troponin T level > 5 times the 99th percentile of the normal upper limit (> 0.07 ng/mL). In total, 57 patients were enrolled, of which 18 (31.6%) had elevation of troponin T > 0.07 ng/mL after PCI and were classified into the PMI group. Significantly worse measurements of IMR (33.7 [24.0–43.8] vs. 26.0 [15.9–33.0], *p* = 0.029) and MRR (2.95 [2.28–4.00] vs. 4.29 [3.01–5.74], *p* = 0.009) were documented in the PMI group. After multivariable logistic regression analyses, both MRR (OR 0.464; 95% CI 0.234–0.919; *p* = 0.028) and IMR (OR 1.060; 95% CI 1.000–1.1830; *p* = 0.041) were identified as predictive factors of PMI. Both MRR and IMR before PCI were independent predictors of PMI.

## Introduction

Periprocedural myocardial injury (PMI) is a common complication of percutaneous coronary intervention (PCI) [1, 2]. Periprocedural myocardial infarction and PMI has been reported to be associated with an increased cardiovascular eventsrisk following PCI [3, 4]; therefore, prevention of PMI is important. Multiple factors are associated with PMI, such as procedure-related, lesion and patient factors [5]. In the absence of a side-branch occlusion or coronary dissection during the procedure, PMI is caused by embolisation into the distal microcirculation, and coronary microvascular dysfunction (CMD) may be associated with reduced resistance to this embolisation [6]. CMD can fundamentally be explained by minimal microvascular resistance and vasodilatory capacity [7]. Minimal microvascular resistance is expressed using the index of microcirculatory resistance (IMR), which is measured invasively [8]. A high IMR, i.e. a high minimal microvascular resistance, was reported to be associated with a high incidence of PMI [9].

The vasodilatory capacity of coronary microvasculature has been evaluated using the coronary flow reserve (CFR) [10]. However, the CFR is reduced in epicardial vessels with stenosis. CFR has limitations as a measure of the vasodilatory capacity of the coronary microvasculature when PCI is performed because studies predicting PMI included patients with stenotic lesions in the epicardial vessels. The microvascular resistance reserve (MRR) was introduced as an index to evaluate microvascular vasodilatory capacity. Unlike the CFR, the MRR is not affected by epicardial lesions [11].

Despite reports on the association between the IMR and PMI, the relationship between microvascular vasodilatory capacity as assessed by MRR and PMI is still unclear. Thus, this study aimed to investigate the association between CMD, consisting of high microvascular resistance (high IMR) and reduced microvascular vasodilatory capacity (low MRR) and PMI assessed by elevation of high-sensitivity troponin T after PCI.

## Materials and methods

### Study population

Between October 2017 and October 2024, 68 patients with stable angina pectoris underwent PCI after measurements of coronary physiological indices at Hiroshima University Hospital. The following patients were excluded from this study: (1) two patients with coronary physiological indices measured in the culprit vessel of prior myocardial infarction; (2) two patients with post-coronary artery bypass grafting; (3) one patient with side-branch occlusion during PCI. The side-branch occlusion was defined as significant reduction in major side branch (> 1.0 mm) blood flow assessed by thrombolysis in myocardial infarction (TIMI) grade; (4) three patients with high troponin T (baseline troponin levels > 99th percentile of the normal upper limit) before PCI; and (5) three patients whose troponin T could not be measured after PCI. At baseline, data on medical history, daily medications, family history of coronary artery disease, blood tests and transthoracic echocardiograms were collected.

This study was approved by the Ethics Committee of Hiroshima University Hospital. The need for written informed consent from the participants was not required owing to the retrospective observational design of this study and the analysis of data collected previously as part of routine clinical care.

### Coronary physiology measurements and PCI procedure

Quantitative coronary angiography was performed using QAngio XA7.3 (Medis Medical Imaging System B.V., Leiden, Netherlands). The minimal lumen diameter, reference vessel diameter, plaque area percentage and each lesion length were quantified using the external diameter of the contrast-filled guiding catheter as the calibration standard. A 5-F guide catheter without a side hole was used to engage the coronary artery. After the intracoronary injection of nitroglycerin (100–200 µg for the right and left coronary arteries), diagnostic coronary angiography was performed. Coronary physiology measurements were performed for lesions with ≥ 50% diameter stenosis and TIMI grade 3 flow. Coronary physiology measurements were performed using a pressure-temperature sensor guidewire (PressureWire × Guidewire Cabled or PressureWire × Guidewire Wireless; Abbott, Santa Clara, CA, USA). The pressure sensor was guided to the distal segment of the target vessel. The resting proximal aortic pressure (P_a,rest_) and distal coronary pressure (P_d,rest_) were measured. To measure the resting mean transit time (T_mn,rest_), thermodilution curves were obtained using three bolus injections (3 mL each) of room-temperature saline. Hyperaemia was induced by continuous intravenous infusion of adenosine (180 µg/kg/min). The hyperaemic proximal aortic pressure (P_a,hyper_) and distal coronary pressure (P_d,hyper_) were also measured. Hyperaemic T_mn_ (T_mn,hyper_) were measured using bolus injections in the same method as at rest. The CFR was calculated by dividing the T_mn,rest_ by T_mn,hyper_. The fractional flow reserve (FFR) was calculated by dividing P_d,hyper_ by P_a,hyper_. The IMR was defined as P_d,hyper_ multiplied by T_mn,hyper_. The MRR, an index of microvascular vasodilatory capacity, was calculated using the following formula [11]:

$${\mathrm{MRR}}=\left( {{\mathrm{CFR}}/{\mathrm{FFR}}} \right) \times \left( {{{\mathrm{P}}_{{\mathrm{a}},{\mathrm{rest}}}}/{{\mathrm{P}}_{{\mathrm{a}},{\mathrm{hyper}}}}} \right)$$

**Fig. 1:**
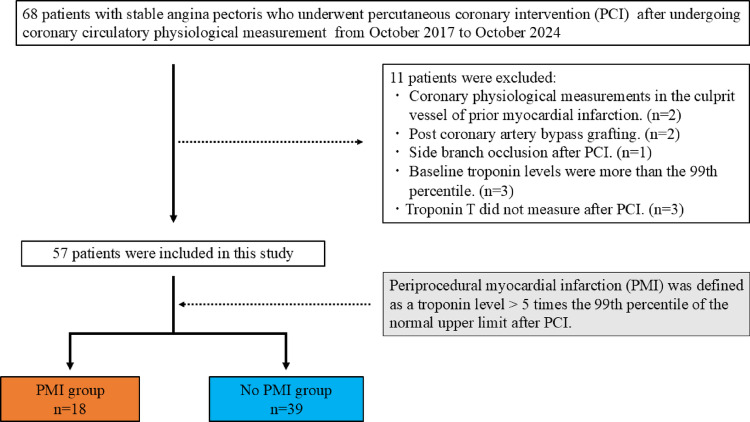
Patient selection flowchart

PCI was performed a median of 32 (17.5–49) days after coronary physiology measurements. PCI was performed for lesions with an FFR < 0.80. The PCI strategy (approach site, mechanical circulatory support and the device to use) was left to the discretion of each clinician. Troponin T was measured 18 h after PCI. In this study, to assess PMI, patients were divided into two groups based on elevation of high-sensitivity troponin T level > 5 times the 99th percentile of the normal upper limit after PCI (troponin T ≥ 0.070 µg/L) [12]. The group with troponin elevation after PCI was defined as the PMI group, and the group without troponin elevation was defined as the no PMI group (Fig. 1).

### Statistical analysis

Continuous variables are presented as means ± standard deviations or median (interquartile range [IQR]). Conversely, categorical variables are presented as numbers and percentages. Comparisons between two groups of continuous variables were performed with the Mann–Whitney U test. Categorical variables were compared using the chi-square or Fisher’s exact test. N-terminal pro-B-type natriuretic peptide (NT-proBNP) values were analysed after natural logarithmic transformation. Univariable and multivariable logistic regression analyses were conducted to examine predictors of PMI. The covariates used in the multivariable analyses were determined using the backward stepwise selection from covariates that reached *p* < 0.05 in the univariable analyses, other than coronary physiology measurements. Multivariable analyses were adjusted using these covariates and MRR or IMR. To evaluate the additional ability of MRR/IMR to predict PMI, C-statistics, net reclassification improvement (NRI), integrated discrimination improvement (IDI) analyses and Akaike information criterion (AIC) were utilised. The clinical baseline model was created from factors used in the multivariate analysis other than the coronary physiological measurements. Model 1 was created by adding MRR to the clinical baseline model, model 2 by adding IMR to the clinical baseline model and model 3 by adding both. C-statistics, NRI and IDI were analysed respectively. A two-sided *p* value < 0.05 was considered significant. All statistical analyses were performed with EZR 1.68 (Saitama Medical Center, Jichi Medical University, Saitama, Japan), which is a graphical user interface for R (The R Foundation for Statistical Computing, Vienna, Austria) [13].

## Results

After exclusion, 57 patients were included, of whom 18 (31.6%) were divided to PMI group. In this cohort, one case of side-branch occlusion was excluded; however, no cases of target vessel occlusion and coronary dissection were documented after PCI. The baseline characteristics of the groups with and without PMI are shown in Table 1, and the characteristics of lesions, procedures and coronary physiological characteristics are shown in Table 2. Patients in the PMI group were older (76.5 [73.0–82.0] vs. 73.0 [65.0–78.0] years, *p* = 0.027) and had a lower body mass index (21.6 [20.7–23.6] vs. 23.9 [21.6–25.3] kg/m^2^, *p* = 0.004). In addition, the PMI group had lower low-density lipoprotein cholesterol level (66.0 [55.3–97.0] vs. 85.0 [73.0–107.5] mg/dL, *p* = 0.018) and a lower rate of statin use (11, 61.1% vs. 35, 89.7%, *p* = 0.026). More patients in the PMI group had longer lesion length (14.27 [12.38–23.17] vs. 9.08 [6.87–10.44] mm, *p* < 0.001), were placed longer stents (33.0 [23.3–47.5] vs. 23.0 [18.0–28.0] mm, *p* = 0.004) and had longer total inflation time (205 [120–250] vs. 155 [105–205] s, *p* = 0.036).

**Table 1:** Baseline characteristics of patients with and without periprocedural myocardial injury

	All *n* = 57	PMI group, *n* = 18	No PMI group, *n* = 39	*p* value
Age, years	74.0 (68.5–79.5)	76.5 (73.0–82.0)	73.0 (65.0–78.0)	0.027
Male	48 (84.2)	14 (77.8)	34 (87.2)	0.442
Body mass index, kg/m^2^	23.6 (21.2–25.0)	21.62 (20.74–23.58)	23.87 (21.62–25.31)	0.004
Hypertension	44 (77.2)	16 (88.9)	28 (71.8)	0.191
Diabetes mellitus	26 (45.6)	9 (50.0)	17 (43.6)	0.777
Dyslipidemia	38 (66.7)	8 (44.4)	30 (76.9)	0.032
Family history of coronary artery disease	15 (26.3)	3 (16.7)	12 (30.8)	0.342
History of smoking	15 (26.3)	4 (22.2)	11 (28.2)	0.753
Chronic kidney disease, (eGFR < 60)	23 (40.4)	10 (55.6)	13 (33.3)	0.150
Atrial fibrillation	19 (33.3)	7 (38.9)	12 (30.8)	0.560
Prior myocardial infraction	11 (19.3)	2 (11.1)	9 (23.1)	0.473
Prior percutaneous coronary intervention	25 (43.9)	7 (38.9)	18 (46.2)	0.775
Hemodialysis	3 (5.3)	2 (11.1)	1 (2.6)	0.232
Medication				
Aspirin	57 (100)	18 (100.0)	39 (100.0)	NA
Clopidogrel	10 (17.5)	5 (27.8)	5 (12.8)	0.260
Prasugrel	47 (82.5)	13 (72.2)	34 (87.2)	0.260
Oral anticoagulant	16 (28.1)	5 (27.8)	11 (28.2)	1.000
βblocker	28 (49.1)	11 (61.1)	17 (43.6)	0.263
ACEi or ARB or ARNI	26 (45.6)	8 (44.4)	18 (46.2)	1.000
Statin	46 (80.7)	11 (61.1)	35 (89.7)	0.026
Ca blocker	30 (52.6)	8 (44.4)	22 (56.4)	0.569
Mineralocorticoid receptor antagonist	5 (8.8)	4 (22.2)	1 (2.6)	0.031
Nitrate	8 (14.0)	1 (5.6)	7 (17.9)	0.414
Nicorandil	4 (7.0)	1 (5.6)	3 (7.7)	1.000
Echocardiography				
Left ventricular ejection fraction, %	61.0 (55.0–66.0)	61.5 (55.5–66.0)	61.0 (56.0,-64.0)	0.957
Left ventricular end-diastolic dimension, mm	48.0 (44.0–51.0)	49.5 (45.5–52.3)	48.0 (44.0-50.5)	0.323
Left ventricular internal dimension in systole, mm	32.0 (29.0–36.0)	33.5 (29.8–36.8)	32.0 (29.0–35.0)	0.476
Left atrial dimension, mm	38.0 (34.8–42.0)	38.0 (36.0-45.5)	38.0 (34.0-41.5)	0.336
Left atrial volume, ml	56.5 (47.6–76.4)	58.2 (51.7–80.8)	56.5 (46.2–67.7)	0.220
Left atrial volume index, ml/m^2^	34.6 (27.9–44.2)	37.4 (32.8–47.1)	33.1 (26.3–37.2)	0.133
Aortic stenosis > moedrate	0 (0)	0 (0)	0 (0)	NA
Aortic regurgitation > moderate	0 (0)	0 (0)	0 (0)	NA
Mitaral regurgitation > moderate	2 (3.5)	2 (11.1)	0 (0.0)	0.096
Tricuspid regurgitation > moderate	4 (7.0)	2 (11.1)	2 (5.1)	0.584
Preprocedural Labo data				
Hemoglobin, g/dL	13.2 (12.1–14.6)	13.0 (11.8–14.3)	13.6 (12.5–14.7)	0.414
Low-density lipoprotein cholesterol, mg/dL	79.0 (64.5-106.5)	66.0 (55.3–97.0)	85.0 (73.0-107.5)	0.018
High-density lipoprotein cholesterol, mg/dL	53.0 (42.5–69.0)	57.5 (44.0–66.0)	53.0 (42.5–68.5)	0.941
Creatinine, mg/dL	0.94 (0.77–1.11)	0.86 (0.74–1.12)	0.96 (0.78–1.04)	0.606
eGFR, mL/min1.73 m^3^	63.0 (50.0-73.5)	59.0 (44.8–74.0)	64.0 (54.0–70.0)	0.612
Log (NT-proBNP)	5.34 (4.42–6.89)	6.17 (5.08–7.43)	5.07 (5.08–7.43)	0.019
CRP, mg/dL	0.07 (0.03–0.17)	0.13 (0.04–0.24)	0.07 (0.02–0.13)	0.284
HbA1c, %	5.95 (5.63–6.80)	6.1 (5.8-7.0)	5.8 (5.7–6.5)	0.406
Creatine kinase, IU/L	90.5 (57.8-125.8)	70.0 (57.3–104.0)	100.5 (69.0-153.3)	0.099
Creatine kinase myocardial band, IU/L	9.0 (8.0–10.0)	9.0 (8.0–10.0)	9.0 (8.0–10.0)	0.810
High sensitive troponin T, ng/mL	0.014 (0.009–0.038)	0.018 (0.012–0.050)	0.012 (0.008–0.026)	0.061

Values are number (%)or medium (interquartile range)

*ACEi* angiotensin converting enzyme inhibitor, *ARB* angiotensin receptor blocker, *ARNI* angiotensin receptor neprilysin inhibitor, *PMI* Periprocedural myocardial injury

**Table 2:** Baseline angiographic, procedural and coronary physiological characteristics

	All *n* = 57	PMI group, *n* = 18	No PMI group, *n* = 39	*p* value
Lesion location				
LMT/LAD/Diag/LCX/RCA	1/48/3/5	0/15/0/1/2	0/33/1/2/3	0.889
QCA analysis				
Reference diameter, mm	2.53 (2.14–2.95)	2.53 (2.19–2.83)	2.53 (2.04–3.01)	0.932
Minimum lumen diameter, mm	1.01 (0.87–1.24)	1.00 (0.87–1.09)	1.03 (0.87–1.25)	0.492
Lesion diameter stenosis, %	58.6 (49.8–64.7)	62.3 (55.4–68.3)	56.7 (49.1–61.2)	0.061
Lesion length, mm	9.85 (7.58–13.14)	14.27 (12.38–23.17)	9.08 (6.87–10.44)	< 0.001
ACC lesion type				
Type A/B1/B2/C	11/22/24/0	0/7/11/0	11/15/13/0	0.017
ACC lesion type > B2	24 (42.1)	11 (61.1)	13 (33.3)	0.082
In stent restenosis	4 (7.0)	1 (5.6)	3 (7.7)	1.000
Procedural				
Drug eluting stent	54 (94.7)	18 (100)	36 (92.3)	0.544
Drug coated balloon	11 (29.3)	5 (27.8)	6 (15.4)	0.297
Pre dilation	49 (86.0)	17 (94.4)	32 (82.1)	0.414
Post dilation	50 (87.7)	18 (100)	32 (82.1)	0.085
Total inflation time	170 (110–235)	205 (120–250)	155 (105–205)	0.036
Longest inflation time	20 (20–25)	20.0 (20.0-27.5)	20.0 (20.0–25.0)	0.968
Maximum inflation pressure	20 (18–22)	20.0 (20.0–22.0)	19.0 (16.5–20.0)	0.035
No. of stents				
0/1/2/3	3/47/6/1	0/14/3/1	3/33/3/0	0.207
Maximum stent diameter	3.0 (2.94–3.5)	3.0 (2.8–3.2)	3.0 (3.0-3.5)	0.381
Total stent length	23 (18–30)	33.0 (23.3–47.5)	23.0 (18.0–28.0)	0.004
Contrast volume	90 (68–110)	100.0 (65.0-125.0)	85.5 (70.0-105.0)	0.518
Intra aortic balloon pumping	1 (1.8)	1 (5.6)	0 (0)	0.316
Debulking device	4 (7.0)	1 (5.6)	3 (7.7)	1.000
Coronary physiology				
FFR	0.72 (0.68–0.76)	0.72 (0.67–0.75)	0.73 (0.69–0.77)	0.335
CFR	2.30 (1.55–3.10)	1.70 (1.33–2.50)	2.80 (1.85–3.55)	0.011
IMR	28.0 (19.2–40.0)	33.7 (24.0-43.8)	26.0 (15.9–33.0)	0.029
MRR	3.60 (2.66–4.96)	2.95 (2.28-4.00)	4.29 (3.01–5.74)	0.009

Values are number (%) or medium (interquartile range)

*ACC* American College of Cardiology, *CFR* Coronary flow reserve, *FFR* Fractional flow reserve, *IMR* Index of microcirculatory resistance, *LAD* left anterior descending artery, *LCX* left circumflex artery, *LMT* Left main trunk, *MRR* Microvascular resistance reserve, *PMI* periprocedural myocardial injury, *QCA* quantitative coronary angiography, *RCA* right coronary artery

**Fig. 2:**
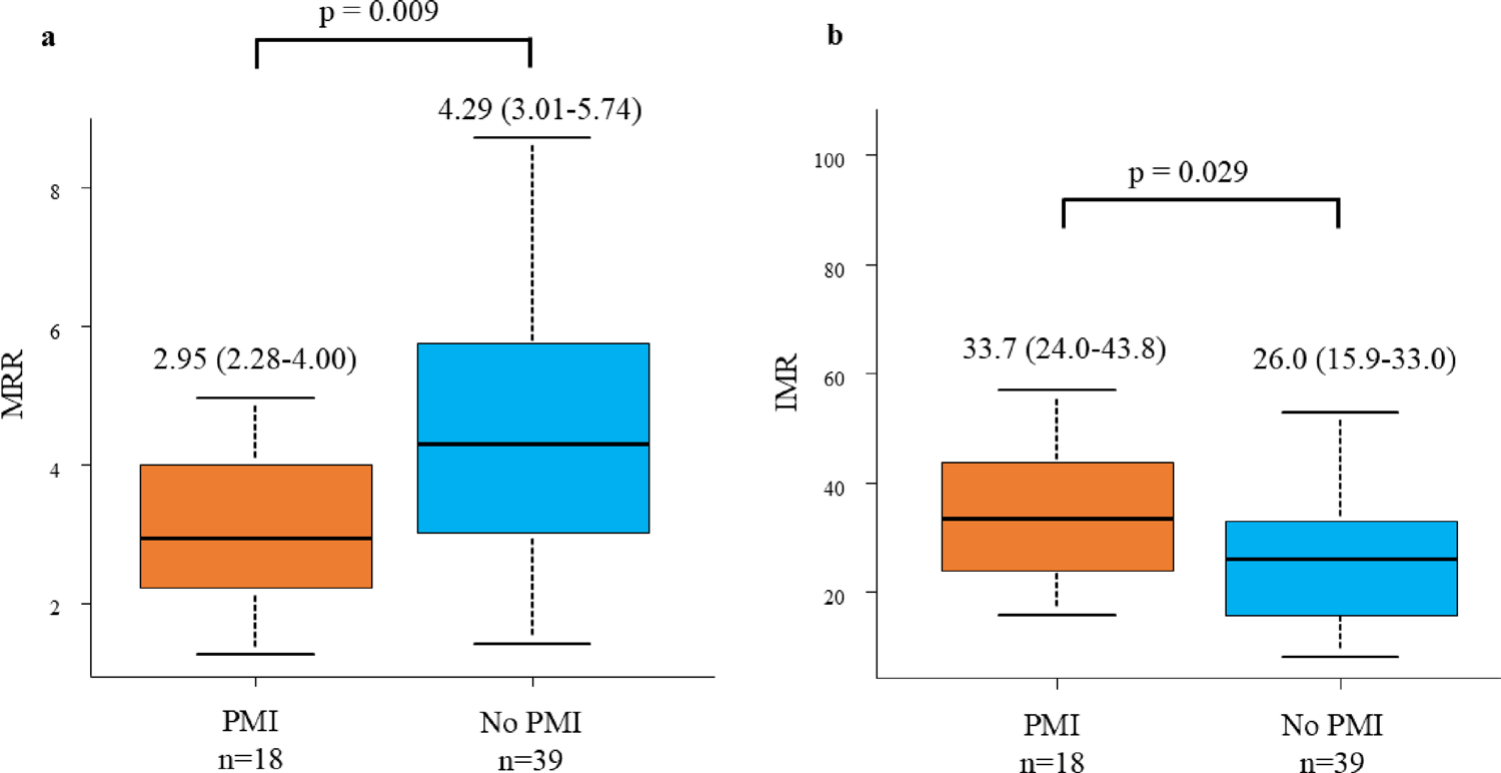
IMR and MRR values before PCI in the PMI and no PMI groups. **a** The IMR value in the PMI group (IMR = 33.7, IQR 24.0–43.8) was higher than that in the no PMI group (IMR = 26.0, IQR 15.9–33.0) (*p* = 0.029). **b** The MRR value in the PMI group (MRR = 2.95, IQR 2.28–4.00) was significantly lower than that in the no PMI group (MRR = 4 0.29, IQR 3.01–5.74) (*p* = 0.009). *IMR* index of microcirculatory resistance, *MRR* microvascular resistance reserve, *PCI* percutaneous coronary intervention, *PMI* periprocedural myocardial injury

No difference was found in the FFR (0.72 [0.67–0.75] vs. 0.73 [0.69–0.77], *p* = 0.331) between the two groups; however, the CFR was lower in the PMI group (1.70 [1.33–2.50] vs. 2.80 [1.85–3.55], *p* = 0.010). When comparing coronary microvascular indices, significantly worse values of IMR (33.7 [24.0–43.8] vs. 26.0 [15.9–33.0], *p* = 0.029) and MRR (2.95 [2.28–4.00] vs. 4.29 [3.01–5.74], *p* = 0.009) were recorded in the PMI group (Fig. 2).

Univariable and multivariable logistic regression analyses were conducted to predict PMI (Table 3). Univariable analysis showed that low CFR (OR 0.422; 95% CI 0.212–0.839; *p* = 0.014), low MRR (OR 0.559; 95% CI 0.354–0.883; *p* = 0.013), high IMR (OR 1.050; 95% CI 1.000–1.100; *p* = 0.014), older age (OR 1.100; 95% CI 1.010–1.190; *p* = 0.026), lower body mass index (OR 0.755; 95% CI 0.603–0.946; *p* = 0.015), higher NT-proBNP levels (OR 1.610; 95% CI 1.080–2.410; *p* = 0.020), non-use of statins (OR 0.180; 95% CI 0.044–0.730; *p* = 0.016), higher max inflation pressure (OR 1.200; 95% CI 1.000–1.440; *p* = 0.046), longer total stent length (OR 1.090; 95% CI 1.030–1.160; *p* = 0.003) and longer total inflation time (OR 1.010; 95% CI 1.000–1.010; *p* = 0.018) were identified as predictors of PMI. In the multivariable analysis with MRR and covariates selected by backward stepwise selection, low MRR (OR 0.464; 95% CI 0.234–0.919; *p* = 0.028), lower body mass index (OR 0.646; 95% CI 0.423–0.987; *p* = 0.043) and longer total stent length (OR 1.160; 95% CI 1.050–1.280; *p* = 0.004) were identified as risk factors of PMI. In the multivariable analysis with IMR, high IMR (OR 1.060; 95% CI 1.000–1.1830; *p* = 0.041), non-use of statins (OR 0.067; 95% CI 0.008–0.539; *p* = 0.011) and longer total stent length (OR 1.160; 95% CI 1.050–1.290; *p* = 0.004) were predictive factors of PMI.

**Table 3:** Univariable and multivariable analysis for periprocedural myocardial injury

	Univariable analysis			Multivariable analysis					
	OR	95% CI	*p* value	OR	95% CI	*p* value	OR	95% CI	*p* value
MRR	0.559	0.354–0.883	0.013	0.464	0.234–0.919	0.028			
IMR	1.050	1.000-1.100	0.031				1.060	1.000-1.130	0.041
CFR	0.422	0.212–0.839	0.014						
Age	1.100	1.010–1.190	0.026						
Body mass index	0.755	0.603–0.946	0.015	0.646	0.423–0.987	0.043	0.709	0.496–1.010	0.059
LDL-C	0.975	0.950–0.999	0.045						
Log (NT-proBNP)	1.610	1.080–2.410	0.020						
Statin	0.180	0.044–0.730	0.016	0.168	0.025–1.150	0.069	0.067	0.008–0.539	0.011
Max inflation pressure	1.200	1.000-1.440	0.046						
Total stent length	1.090	1.030–1.160	0.003	1.160	1.050–1.280	0.004	1.160	1.050–1.290	0.004
Total inflation time	1.010	1.000-1.010	0.018						

Multivariable analyses were performed with MRR or IMR, respectively and variables that selected by backward stepwise selection

*CFR* Coronary flow reserve, *IM*R Index of microcirculatory resistance, *LDL-C* Low-density lipoprotein cholesterol, *MRR* microvascular resistance reserve

Table 4 shows the results of the AIC, c-statistic, NRI and IDI conducted to assess the predictive ability of PMI, with the addition of coronary microcirculation measurements to the clinical baseline model. The clinical baseline model included body mass index, statin use and total stent length. The model added with MRR, IMR, or MRR and IMR was compared with the clinical baseline model. When comparing the model that included MRR with the clinical baseline model, although the c-statistics was not significant, the NRI (0.829; 95% CI 0.324–1.334; *p* = 0.001) and IDI (0.091; 95% CI 0.017–0.166; *p* = 0.017) values improved, indicating that MRR showed incremental predictive ability of PMI. Similarly, when comparing the model that included IMR with the clinical baseline model, both NRI (0.913; 95% CI 0.182–1.237; *p* = 0.008) and IDI (0.100; 95% CI 0.002–0.198; *p* = 0.045) values improved. Furthermore, the addition both the MRR and IMR improved the NRI (0.974; 95% CI 0.477–1.472; *p* < 0.001) and IDI (0.141; 95% CI 0.041–0.242; *p* = 0.006) values, and the AIC of this model was the smallest among all the models analysed.

**Table 4:** Predictive ability of MRR and IMR in addition to clinical baseline model for periprocedural myocardial injury

Model	AIC	C-statistic	*p* value	NRI	95% CI	*p* value	IDI	95% CI	*9* value
Clinical baseline model	52.393	0.880	Reference		Reference			Reference	
Clinical baseline model + MRR	47.825	0.909	0.266	0.829	0.324–1.334	0.001	0.091	0.017–0.166	0.017
Clinical baseline model + IMR	48.044	0.913	0.141	0.709	0.182–1.237	0.008	0.100	0.002–0.198	0.045
Clinical baseline model + MRR + IMR	46.792	0.922	0.130	0.974	0.477–1.472	< 0.001	0.141	0.041–0.242	0.006

Clinical baseline model = body mass index, total stent length and statin

*AIC* Akaike information criterion, *IDI* integrated discrimination improvement, *IMR* Index of microcirculatory resistance, *MRR* Microvascular resistance reserve, *NRI* Net reclassification improvement

## Discussions

The main finding of this study was that the MRR before PCI, which is an index of the vasodilatory capacity of the coronary microvasculature, was a predictor of PMI. The MRR and IMR remained a predictor of PMI following multivariate analysis. Prediction models that added MRR or IMR to the clinical baseline model (body mass index, statin use and total stent length) had improved predictive ability for PMI compared with models not added with these metrics. To our knowledge, no previous studies have examined the relationship between MRR before PCI and PMI.

It has been reported that periprocedural myocardial infarction is associated with an increased risk of all-cause mortality after PCI [3]. Periprocedural myocardial infarction is diagnosed by periprocedural elevation of cardiac biomarker such as troponin, as well as cillary criteria suggestive of myocardial ischemia such as electrocardiogram changes or imaging findings [12]. PMI is assessed by periprocedural troponin elevation and one of the diagnostic criteria for periprocedural myocardial infarction. PMI in patients with stable angina has also been reported to be associated with an increase in cardiac events after PCI [4]. Therefore, the identification of the risk factors of PMI may help prevent its development and improve patient prognosis. Two mechanisms have been proposed for PMI: dissection or side-branch occlusion near the lesion to be treated and structural and functional distal occlusion [5]. The latter accounts for 50–70% of PMI [5]. Distal occlusion is caused by the embolic material produced by atherosclerotic plaque disruption or local vascular trauma, which can occlude both epicardial vessels and coronary microvasculature [5]. CMD may induce plugging and decrease the clearing of embolic materials, increasing the susceptibility to PMI development [9].

### Association of CMD with PMI

Two CMD pathologies have been proposed: minimal microvascular resistance and vasodilatory capacity of the coronary microvasculature [7]. The IMR, a measure of minimal microcirculatory resistance, was reported to be a predictor of PMI. Martin K.C. Ng et al. reported that a high IMR before PCI was a predictor of PMI defined by elevated troponin levels > 3 times the 99th percentile of the normal upper limit after PCI [9]. That study included only the left anterior descending artery, whereas the present study included all coronary arteries. Nevertheless, after the multivariable analysis, the IMR was identified as a significant predictor of PMI (OR 1.060; 95% CI 1.000–1.130; *p* = 0.041).

The vasodilatory capacity of the coronary microvasculature was previously assessed by the CFR because the CFR can be an indicator of vasodilatory capacity in the absence of coronary artery stenosis [10]. Albertal et al. reported that the coronary flow velocity reserve after PCI was a predictor of PMI development; however, they did not report a direct association between the coronary flow velocity reserve before PCI and PMI [14]. To prevent PMI, the risk needs to be understood before conducting PCI. However, given that the CFR also evaluates stenosis of epicardial vessels, it is not a pure measure of CMD in patients with angina pectoris who have residual stenosis. Therefore, the CFR has limitations as an index for investigating the association between CMD and PMI. Recently, the MRR, an index independent of epicardial disease, was proposed to assess the vasodilatory capacity of the coronary microvasculature [11]. The MRR is defined as microvascular resistance at rest divided by microvascular resistance at maximal hyperaemia, under the assumption that epicardial arteries are normal. In actual measurements, MRR corrects CFR for the effects of epicardial stenosis and systemic hemodynamics through division by FFR and correction for blood pressure fluctuations due to hyperemia, thereby providing a more specific measure of microvascular vasodilatory capacity. A low MRR was reported to be useful for CMD diagnosis [15], and low MRR was associated with a poor prognostic factor after PCI for stable coronary artery disease [16] and acute coronary syndrome [17]. Furthermore, Nishi et al. reported a higher incidence of PMI in the group with lower MRR after PCI for stable coronary artery disease [16].

In these previous studies, low CFR and MRR after PCI, indices of vasodilatory capacity of the coronary microvasculature, have shown an association with PMI. In addition, patients with baseline CMD were more susceptible to postprocedural coronary flow velocity reserve impairment [14]; therefore, the presence of CMD at baseline may be potentially associated with a lack of functional reserve to additional impairment. In this study, the MRR before PCI was identified as a predictor of PMI, indicating that the poor vasodilatory capacity of the coronary microvasculature at baseline may be associated with PMI.

This study was unable to clarify whether PMI was associated with cardiovascular events beyond the periprocedural period. However, PMI in patients with stable angina has previously been reported to be associated with an increased risk of cardiac events [4]. Sakamoto et al. reported that an increase in angiography-derived IMR before and after PCI using rotational atherectomy was associated with the occurrence of cardiovascular adverse events after PCI [18]. Both PMI and periprocedural CMD worsening have been reported in previous studies to be associated with cardiovascular adverse prognosis, warranting consideration of these associations. Previous studies have reported that MRR after PCI was associated with PMI [16]. In contrast, this study found that MRR before PCI were associated with PMI. This finding, which allows for the prediction of PMI risk prior to procedure, may be considered useful for prevention of PMI and improving the prognosis of patients after PCI.

Many predictors of PMI have been reported, including patient factors such as age, diabetes, body mass index, multivessel disease, long lesion length, systemic atherosclerosis, pre-existing chronic kidney disease, anaemia, high pre-procedural CRP, statin use and procedural factors such as device selection, number of stents, balloon inflation time, side-branch occlusion and dissection [6, 19]. In this study, a low body mass index, statin use and long stents were identified as predictors of PMI. This result was consistent with the findings of previous studies that lesion complexity and instability were associated with PMI. Statin use contributes to PMI prevention by improving plaque stability [20], and longer stents are needed for more complex lesions and are associated with procedural complexity.

In this study, the PMI group had lower rates of statin use and lower low-density lipoprotein cholesterol levels. Statin use is an established tool for PCI risk reduction, including PMI prevention and routine administration before PCI is recommended [21]. In a meta-analysis of 13 randomised studies, high-dose statin use before PCI reduced a risk of PMI, defined as postintervention creatine kinase-myocardial band increase > 3 times the upper limit of normal [22]. Plaque stabilisation has been considered the mechanism by which statin use prevents PMI. However, in an analysis of the relationship between IMR before PCI and PMI, Martin et al. reported that patients using statins had lower IMR than those who were not using statins [9]. Statins have not only cholesterol-lowering effects but also pleiotropic effects, such as vasodilation, inflammation inhibition and improvement of endothelial function, which contribute to the improvement of microcirculation function [23]. In this study, the PMI group with low statin use had higher IMR and lower MRR, indicating that statin use may contribute to the improvement in microcirculation and may be associated with PMI prevention. Conversely, in the ‘The Impact of evolocumab on periprocedural microvascular damage in patients undergoing PCI (EVOCATION)’ trial, Ishihara et al. reported that although the additional administration of evolocumab to statin therapy to patients with chronic coronary syndrome significantly reduced low-density lipoprotein cholesterol levels, no difference was observed in the IMR before PCI and troponin T levels after PCI [24]. Thus, the pleiotropic effect of statin, rather than their low-density lipoprotein cholesterol-lowering effect, may be more useful for improving microcirculation and preventing PMI. In the present study, this factor was considered to influence the higher incidence of PMI in patients who were not prescribed statins because their low levels of low-density lipoprotein cholesterol. The statin use before PCI may offer not only plaque stabilization but also promising effects in preventing PMI through improved CMD. Further study is needed on preventive measures for PMI, including statin use.

The PMI group had higher NT-proBNP levels than the other group. Murai et al. examined the effect of coronary microcirculation on the prognosis of patients without coronary artery disease and reported that high BNP levels were associated with high IMR or low CFR [25]. Dryer et al. also reported that a higher proportion of patients with heart failure with preserved ejection fraction had either abnormal IMR or CFR compared with healthy controls [26]. Katsura et al. reported that CMD was directly related to left atrial remodeling and may be associated with left ventricular diastolic dysfunction [27]. Moreover, worsening coronary microcirculation due to heart failure may be one of the factors contributing to PMI. However, in the present study, although NT-proBNP was a predictive factor of PMI in the univariable analysis, it was not a significant predictive factor in the multivariable analysis.

This study showed that low MRR before PCI was an independent predictor of PMI, even after multivariable analysis accounting for other variables predicting PMI. In addition, the predictive model added with MRR and IMR was more useful in predicting PMI, increasing both NRI and IDI compared with the baseline clinical model that included only body mass index, statin use and total stent length. In other words, the predictive performance for PMI was improved when also adding MRR, an index specific to the vasodilatory function of the microvasculature and IMR, an index of microvascular resistance, to indices representing the complexity and lesion characteristics of epicardial lesions. CMD before PCI, assessed by MRR and IMR, may be an independent risk factor for PMI.

### Study limitations

This study has several limitations. First, this was a single-centre, retrospective, observational study of Japanese patients. The selection of patients and the exclusion criteria may have introduced selection bias. Second, the sample size was relatively small because this study was a single center retrospective study. Therefore, the results of this study should be interpreted with caution. Third, the MRR was based on bolus thermodilution. The bolus thermodilution method may result in slightly higher MRR values than when measured by continuous thermodilution; however, the diagnostic and prognostic values of MRR appear to be maintained even if calculated using alternative methodologies [28]. Fourthly, despite coronary physiologic measurements on patients with stenotic coronary artery disease, the influence of collateral flow was not considered in the IMR calculations. Therefore, IMR measurements may be overestimated. Fifth, this study did not analyse intravascular imaging of lesions subjected to PCI. Differences in plaque characteristics may affect the incidence of PMI. Sixth, we did not perform coronary physiology measurements after PCI. Therefore, we were unable to evaluate changes in MRR before and after PCI. Lastly, we were unable to use information on follow-up events such as cardiovascular events, rehospitalization, and mortality, and therefore could not assess the direct impact of PMI on prognosis. Further studies are needed to clarify how MRR before PCI induces PMI and how affects the prognosis after PCI.

## Conclusion

In this study, the MRR before PCI was identified as an independent predictor of PMI. This finding suggests that a low MRR before PCI may contribute to the risk of PMI. Further studies are needed to elucidate the causes of low MRR and therapeutic strategies to improve MRR to prevent PMI and improve outcomes after PCI.
